# PCSK9 inhibitor and atorvastatin reduce cardiac impairment in ovariectomized prediabetic rats via improved mitochondrial function and Ca^2+^ regulation

**DOI:** 10.1111/jcmm.15556

**Published:** 2020-07-06

**Authors:** Patchareeya Amput, Siripong Palee, Busarin Arunsak, Wasana Pratchayasakul, Chanisa Thonusin, Sasiwan Kerdphoo, Thidarat Jaiwongkam, Siriporn C. Chattipakorn, Nipon Chattipakorn

**Affiliations:** ^1^ Cardiac Electrophysiology Research and Training Center Faculty of Medicine Chiang Mai University Chiang Mai Thailand; ^2^ Cardiac Electrophysiology Unit Department of Physiology Faculty of Medicine Chiang Mai University Chiang Mai Thailand; ^3^ Center of Excellence in Cardiac Electrophysiology Research Chiang Mai University Chiang Mai Thailand; ^4^ Department of Physical Therapy Faculty of Allied Health Science University of Phayao Phayao Thailand

**Keywords:** atorvastatin, heart, insulin resistance, obesity, oestrogen deprivation, PCSK9 inhibitor

## Abstract

Post‐menopausal women have a higher risk of developing cardiometabolic dysfunction. Atorvastatin attenuates dyslipidaemia and cardiac dysfunction but it can have undesirable effects including increased risk of diabetes and myalgia. Currently, the proprotein convertase subtilisin/kexin type 9 (PCSK9) inhibitor efficiently reduces low‐density lipoprotein cholesterol (LDL‐C) levels more effectively than atorvastatin. We have been suggested that PCSK9 inhibitor attenuated cardiometabolic impairment more effectively than atorvastatin in ovariectomized prediabetic rats. Female Wistar rats (n = 48) were fed a normal diet (ND) or high‐fat diet (HFD) for 12 weeks. Then, HFD rats were assigned to a sham‐operated (Sham) or ovariectomized (OVX) group. Six weeks after surgery, the OVX group was subdivided into 4 treatment groups: vehicle (HFOV), atorvastatin (HFOA) (40 mg/kg/day; s.c.), PCSK9 inhibitor (HFOP) (4 mg/kg/day; s.c.) and oestrogen (HFOE_2_) (50 µg/kg/day; s.c.) for an additional 3 weeks. Metabolic parameters, cardiac and mitochondrial function, and [Ca^2+^]_i_ transients were evaluated. All HFD rats became obese‐insulin resistant. HFS rats had significantly impaired left ventricular (LV) function, cardiac mitochondrial function and [Ca^2+^]_i_ transient dysregulation. Oestrogen deprivation (HFOV) aggravated all of these impairments. Our findings indicated that the atorvastatin, PCSK9 inhibitor and oestrogen shared similar efficacy in the attenuation in cardiometabolic impairment in ovariectomized prediabetic rats.

## 1 INTRODUCTION

Cardiovascular disease (CVD) is the major leading cause of death worldwide, this incidence being expected to continue until 2030 on current projection alone.^1^ It is well known that long‐term exposure to a high‐fat diet (HFD) causes obesity and insulin resistance.^2^ Insulin resistance is a proven risk for CVD because of its adverse effects on blood pressure regulation, fat metabolism, blood coagulation and arterial system.^3^, ^4^ Although there is a lower frequency of CVD events in women in comparison with men at the same age, the incidence increases during menopause.^5^ Women who had had a bilateral ovariectomy (OVX) had an increase in mortality from CVD; however, it has been shown that an oestrogen supplement could reduce the risk showing the beneficial effects of oestrogen on the heart.^6^ An increase in blood pressure has been identified in menopausal women when compared with age‐matched men, suggesting oestrogen deprivation has a role in hypertensive women.^7^ Following menopause, women exhibit both an increased cardiovascular risk and an increased prevalence of metabolic syndrome including visceral fat deposition, central obesity, dyslipidaemia and insulin resistance.^8^ In addition to the positive effects on the cardiovascular system, oestrogen administration in OVX rats has been shown to improve metabolic function, especially by enhancing insulin sensitivity.^9^ However, as long‐term oestrogen treatment increases the risks of breast cancer and ovarian cancer in post‐menopausal women,^10^ an alternative treatment with equally effective cardiometabolic protection is preferable for post‐menopausal women.

Lipid‐lowering drugs have been used to decrease the cardiovascular risk in post‐menopausal women.^11^ One such drug atorvastatin inhibits the HMG‐CoA reductase enzyme, thus decreasing the biosynthesis of cholesterol.^11^ In addition to its lipid‐lowering effect, atorvastatin therapy showed enhancement of glucose metabolism and insulin sensitivity,^12^ and also reduction of cardiac oxidative stress in obese rats.^13^, ^14^ However, several clinical studies have reported an inadequate decrease in cardiovascular risks associated with atorvastatin due to inadequate reduction in low‐density lipoprotein cholesterol (LDL‐C) level.^15^, ^16^ Also, long‐term use of atorvastatin showed a correlation with a higher risk of diabetes and myalgia.^17^, ^18^ Currently, a novel lipid‐lowering drug a proprotein convertase subtilisin/kexin type 9 (PCSK9) inhibitor^19^ has been shown to effectively reduce the LDL‐C levels in the blood via reducing the degradation of low‐density lipoprotein receptors (LDLR).^20^ More recently, clinical cardiovascular outcome trials with the PCSK9 inhibitor revealed a significant decrease in the risk of CVD.^21^, ^22^ However, there is no research to show the effects of the PCSK9 inhibitor on cardiac function or to investigate the comparative effects of PCSK9 inhibitor and atorvastatin on cardiac function, mitochondrial function and intracellular Ca^2+^ transient in the ovariectomized prediabetic condition. In this study, we have been suggested that the PCSK9 inhibitor attenuates metabolic impairment and cardiac dysfunction more effectively than atorvastatin in ovariectomized prediabetic rats.

## 2 MATERIALS AND METHODS

### 2.1 Ethical approval and animal preparation

Experimental protocols were performed in accordance with the Guide for the Care and Use of Laboratory Animals published by the NIH and approved by the Laboratory Animal Center, Chiang Mai University and Chiang Mai University Animal Care and Use Committee, Chiang Mai University (approval no. 2561/RT‐0002).

Forty‐eight female Wistar rats (weighing 200‐220 g, 6 weeks old) were acquired from the Nomura Siam International Co, Ltd., Bangkok, Thailand. The rats were kept in a controlled temperature room for acclimatization process for 1 week; then, they were randomly allocated into two groups.

### 2.2 Experimental protocol

The normal diet (ND, a diet containing 19.77% E fat, n = 8) and a high‐fat diet (HFD, a diet containing 59.28% E fat, n = 40) were given to the rats for 12 weeks.^23^ At the end of the 12‐week period, the ND and HFD rats were divided into the sham‐operated (Sham, n = 8), including NDS, which is normal diet‐fed sham‐operated rats and HFS, which is high‐fat diet‐fed sham‐operated rats or bilateral ovariectomy (OVX) (n = 32) groups. Six weeks after surgery, the OVX group was subdivided into 4 treatment groups (n = 8/group), including HFOV, which is high‐fat diet‐fed ovariectomized rats (normal saline; s.c.), HFOA, which is high‐fat diet‐fed ovariectomized rats treated with atorvastatin (40 mg/kg/day; s.c.), HFOP, which is high‐fat‐diet fed ovariectomized rats treated with PCSK9 inhibitor (SBC‐115076, 4 mg/kg/day; s.c.) and HFOE_2_, which is high‐fat diet‐fed ovariectomized rats treated with oestrogen (50 µg/kg/day; s.c.) for 3 additional weeks. Throughout the experimental period, food intake and bodyweight were recorded. After 3 weeks of treatment, metabolic and cardiac parameters were determined. One day after the completion of treatment, a pressure‐volume (P‐V) loop was used to investigate cardiac function. Subsequently, the rats were decapitated an each rat's heart was removed to enable the investigation of the cardiac mitochondrial function, oxidative stress, apoptosis and intracellular Ca^2+^ transient.

### 2.3 Ovariectomy procedure

A combination of Xylazine (0.15 mL/kg) and Zoletil (50 mg/kg) was used for anesthetizing the rats. After deep anaesthesia was confirmed by a loss of righting reflex, a midline dorsal incision was made between the front of the hind limb and the bottom of the rib cage. The blood vessel was squeezed, and the uterine horn and uterine tube were dissected, and the ovaries were removed. Then, the wound was closed by suturing the abdominal wall. After the operation, rats were given antibiotics including marbocyl and tolfedine.^24^ Then, the rats were placed in a clean dry bedded box for 1 week.

### 2.4 Assessment of metabolic markers

Plasma glucose, LDL, triglyceride and HDL levels were assessed using commercially available kits (Biotech, Bangkok, Thailand). Insulin levels were determined using a commercial sandwich ELISA kit (Millipore, Burlington, MA, USA).^25^ The severity of insulin resistance was indicated by areas under the curve (AUC) following OGTT and homeostasis model assessment (HOMA) index.^26^

### 2.5 Determination of blood pressure

Blood pressures were determined using a non‐invasive CODA volume‐pressure monitoring system (Kent Scientific Corporation, Torrington, CT, USA). Rats had mobility limited using a restrainer, and the tails were attached to occlusion (O‐cuff) and volume‐pressure recording (VPR) sensors.^27^

### 2.6 Echocardiography

An echocardiograph (GE vivid‐i, GE healthcare, Chicago, IL, USA) was used to assess LV function. Light anaesthesia was induced in the rats using isoflurane (2%) with oxygen (2 L/min). The LV papillary muscle level was located to determine M‐mode echocardiographic images. Fractional shortening (%FS) was measured.^27^ The diastolic function was determined using the E/A ratio detected from the colour Doppler from an apical four‐chamber view.^27^

### 2.7 Determination of heart rate variability (HRV)

Electrocardiograms (lead II) were carried out (Power Lab 4/25 T, AD Instruments, Sydney, NSW, Australia) in each rat to determine HRV and were fed through a Chart 5.0 program (AD Instruments) for 20 minutes. The HRV data were analysed using the MATLAB program. The parasympathetic tone was indicated by high frequency (HF) in the range 0.15‐0.40 Hz, whereas a low frequency (LF) in the range 0.04‐0.15 Hz was used to represent parasympathetic and sympathetic tone.^28^ Cardiac sympathetic/parasympathetic balance was represented by the LF/HF ratio. A high LF/HF ratio represents an impaired cardiac sympathovagal balance.^2^, ^28^

### 2.8 Determination of LV function by pressure‐volume (P‐V) loop

A combination of Xylazine (0.15 mg/kg) and Zoletil (50 mg/kg) was used to anaesthetize the rats via intramuscular injection. For the determination of LV pressure and volume, the insertion of the P‐V loop catheter was directed to the LV chamber. After stabilization for 10 minutes, the signalling data from all loops were recorded for 20 minutes. Labscribe software analysis (Dover, NH, USA) was used to analyse, maximum and minimum d*P*/d*t* (d*P*/d*t*_max_ and d*P*/d*t*_min_), end‐systolic and diastolic pressure (ESP and EDP), cardiac output (CO), ejection fraction (EF) and stroke work (SW). In addition, heart rate values, left ventricular end‐systolic volume (LVESV) and left ventricular end‐diastolic volume (LVEDV) were determined using the same methods used in LV function measurement.^27^, ^29^

### 2.9 Determination of cardiac mitochondrial function

The cardiac mitochondrial ROS level, cardiac mitochondrial membrane potential changes and cardiac mitochondrial swelling were measured using the methods reported previously.^29^ The heart was immediately removed for the isolation of cardiac mitochondria as previously described.^29^ Dichlorohydrofluoresce in diacetate (DCFDA) dye was put into the isolated cardiac mitochondria for 20 minutes. The ROS production was determined with a fluorescent microplate reader (BioTek Instruments) with an excitation at 485 nm and emission at 530 nm.^30^ JC‐1 dye was put into isolated cardiac mitochondria for 30 minutes. Then, the change in cardiac mitochondrial membrane potential was investigated using a JC‐1 monomer, which recognized green fluorescence (at excitation/emission: 485/590 nm), and JC‐1 aggregates, which recognized red fluorescence (at excitation/emission: 485/530 nm). Mitochondrial membrane depolarization was presented as a reduction in red/green ratio.^31^ A decreased absorbance indicates cardiac mitochondrial swelling in a mitochondrial suspension (540 nm) and was determined using a spectrophotometer.^32^, ^33^ A transmission electron microscope (TEM; JEM‐1200 EX II, JEOL Ltd.,Tokyo, Japan) was used to evaluate any abnormality in cardiac mitochondrial morphology.

### 2.10 Determination of cardiac mitochondrial dynamics

The numerous levels of protein expression were measured by Western blot method. The proteins included dynamin‐related protein 1 (Drp1), mitofusin 1 (Mfn1), mitofusin 2 (Mfn2), optic atrophy 1 (OPA1) and peroxisome proliferator‐activated receptor gamma coactivator 1 (PGC1). The protein from cardiac tissue lysates was added to a loading buffer (5% of mercaptoethanol, 0.05% of bromophenol blue, 75 nmol/L of Tris, 2% of SDS and 10% of glycerol with pH 6.8). The mixture was then heated at 95°C for 5 minutes. The mixture was loaded into 10% gradient SDS‐polyacrylamide gels, and the protein was transferred to a nitrocellulose membrane with a glycine/methanol transfer buffer. Membranes were incubated in 5% skim milk or bovine serum albumin (BSA) for 1 hours, and exposed to anti‐phospho‐Drp1 (ser616), total‐Drp1, Mfn1, Mfn2, OPA1, PGC1 and VDAC (Cell Signaling Technology) for 12 hours. Horseradish peroxidase merged with antirabbit IgG was used to detect bound antibody, and membranes were exposed to enhanced chemiluminescence (ECL).

### 2.11 Measurement of cardiac MDA concentration

A high‐performance liquid chromatography (HPLC) system (Thermo Scientific, Bangkok, Thailand) was used to evaluate malondialdehyde (MDA) levels in cardiac tissues.^34^ Total protein was combined with 10% trichloroacetic acid (TCA) containing BHT then heated at 90°C for 30 minutes and cooled down to room temperature. The mixture was centrifuged, and the supernatant was added to 0.44 M H_3_PO_4_ and 0.6% thiobarbituric acid (TBA) solution to generate thiobarbituric acid reactive substances (TBARS). The concentration of TBARS was assessed using an HPLC system.

### 2.12 Cardiac apoptotic expression and lipid metabolism proteins

The numerous levels of protein expressions were measured by Western blot method. Anti‐Bax, Bcl‐2, Caspase 3, Cleaved‐ Caspase 3, anti‐cytochrome *c* (Cyt *c*) (Cell Signaling Technology, Danvers, MA, USA), PCSK9 (1:1000 dilution, Abcam, Cambridge, UK), LDLR (1:200 dilution, Abcam, Cambridge, UK) and anti‐actin (Sigma‐Aldrich, St. Louis, MO, USA) were used. In addition, the expressions of PCSK9, LDLR, CPT1 and complex I–V in oxidative phosphorylation (OXPHOS) in the liver were determined using the same methods used in cardiac tissues. The detection of horseradish peroxidase merged with antirabbit IgG was used to detect the bound antibody, and membranes were exposed to enhanced chemiluminescence (ECL).^29^

### 2.13 TUNEL‐positive apoptotic cells

A TUNEL assay kit (Roche, Basel, Switzerland) was used to assess the level of cardiomyocyte apoptosis. After dehydration, the cardiac tissue slices were put in 1X PBS for 10 minutes in situ labelling, and the samples were coated with 50 μL of Proteinase k solution (1:50) for 30 minutes followed by 50 μL of Cytonin™ for 120 minutes. The samples were coated with TACS nuclease 1:50 in TACS nuclease buffer. The number of TUNEL‐positive cells was recorded using fluorescence microscopy (Nikon) at λ_ex_ 494 nm and λ_em_ 512 nm. DAPI was detected at λ_ex_ 358 nm and λ_em_ 461 nm. A percentage of the TUNEL‐positive apoptotic cells number over the total number of nucleated cells (DAPI staining) gave the apoptosis index.^35^

### 2.14 Intracellular Ca^2+^ transient measurements

The Ca^2+^ transient level in cardiomyocytes was measured using a fluorescent Ca^2+^ indicator; Fura‐2‐AM at 25 µmol/L (Sigma Chemical, St. Louis, MO, USA) was added to the cardiomyocytes for 30 minutes in the incubator at room temperature. Ultraviolet light (340 and 387 nm) and a monochromator were used to excite the Fura‐2 which was regulated using a microfluorometry system (Cell^R^, Olympus, Tokyo, Japan); then, the light of excitation was conducted into an inverted microscope (IX‐81; Olympus). The emitted fluorescence signal ratio from the Fura‐2‐AM at 510 nm was detected. The Ca^2+^ transient parameters including the Ca^2+^ transient amplitude, rising and decay rate and the diastolic Ca^2+^ levels were determined during a 1‐Hz field‐stimulation with supra‐maximal threshold strength square‐wave pulses (10 ms). Xcellence imaging software (Olympus) was used to determine the fluorescence intensity ratio.^29^

### 2.15 Statistical analysis

Data were presented as mean ± SEM. A one‐way ANOVA followed by an LSD post hoc test was used to compare the variables. The statistical significance was considered at *P* < 0.05.

## 3 RESULTS

### 3.1 Atorvastatin, PCSK9 inhibitor and oestrogen treatments similarly decreased metabolic impairment in ovariectomized prediabetic rats

After 21 weeks of HFD consumption, all HFD rats developed obese‐insulin resistance, indicated by an impaired glucose tolerance test, when compared to NDS rats (Table 1). These impairments were worse in HFOV rats in comparison with HFS rats (Table 1). Interestingly, after 3 weeks of atorvastatin (HFOA), PCSK9 inhibitor (HFOP) and oestrogen (HFOE_2_) treatment, rats in these groups had significantly reduced bodyweight and visceral fat, when compared to HFOV rats. However, HFOE_2_ rats had a greater reduction in bodyweight and visceral fat than HFOA and HFOP rats (Table 1). Insulin sensitivity was similarly improved in HFOA, HFOP and HFOE_2_ rats, compared to HFOV rats, indicated by a markedly reduced area under the curve (AUC) in the OGTT (Table 1). HFOA, HFOP and HFOE_2_ rats had significant increases in fasting blood glucose level, compared with NDS rats (Table 1). Rats in all treatment groups had no significant difference in food intake (Table 1). According to lipid profiles, HFOA, HFOP and HFOE_2_ rats had significantly reduced plasma cholesterol levels and plasma LDL levels, compared to HFOV rats (Table 1). However, plasma triglyceride levels were significantly reduced in HFOA and HFOP rats, compared to HFS and HFOV rats, whereas HFOE_2_ rats had significantly increased plasma triglyceride levels, when compared to HFOA and HFOP rats (Table 1).

**Table 1:** Effects of atorvastatin, PCSK9 inhibitor and estrogen on metabolic parameters in ovariectomized prediabetic rats

Parameters	Groups					
	NDS	HFS	HFOV	HFOA	HFOP	HFOE_2_
Bodyweight (g)	266.59 ± 5.21	347.66 ± 5.90*	399.70 ± 10.41*’^†^	357.15 ± 10.69*’^‡^	356.04 ± 3.01*’^‡^	313.66 ± 6.27*’^†^’^‡^’^#^’^$^
Visceral fat (g)	10.69 ± 0.72	25.90 ± 1.31*	33.88 ± 2.61*’^†^	25.44 ± 2.23*’^‡^	24.74 ± 1.26*’^‡^	18.96 ± 0.99*’^†^’^‡^’^#^’^$^
Uterus weight (g)	0.58 ± 0.06	0.58 ± 0.04	0.14 ± 0.01*’^†^	0.12 ± 0.01*’^†^	0.16 ± 0.01*’^†^	0.29 ± 0.05*’^†^’^‡^’^#^’^$^
Estradiol level (pg/mL)	134.78 ± 10.12	132.70 ± 13.82	20.01 ± 10.22*’^†^	15.74 ± 2.67*’^†^	16.08 ± 4.66*’^†^	83.57 ± 24.50*’^†^’^‡^’^#^’^$^
Food intake (g/day)	15.99 ± 0.57	16.12 ± 0.22	16.53 ± 0.46	16.53 ± 0.34	16.44 ± 0.53	16.11 ± 0.30
Glucose (mg/dL)	154.92 ± 7.22	231.13 ± 9.74*	261.26 ± 8.07*’^†^	227.16 ± 8.15*’^‡^	221.28 ± 10.21*’^‡^	226.25 ± 2.24*’^‡^
Insulin (ng/mL)	2.00 ± 0.22	4.78 ± 0.30*	7.95 ± 1.02*’^†^	3.87 ± 0.50*’^‡^	4.03 ± 1.01*’^‡^	4.07 ± 0.62*’^‡^
HOMA index	19.21 ± 2.56	61.22 ± 8.42*	103.91 ± 13.41*’^†^	53.96 ± 6.05*’^‡^	55.06 ± 16.04*’^‡^	55.65 ± 7.40*’^‡^
Plasma glucose AUC (AUCg) (mg/dL × min × 10^4^)	0.71 ± 0.08	1.83 ± 0.13*	2.91 ± 0.06*’^†^	1.60 ± 0.12*’^‡^	1.60 ± 0.30*’^‡^	1.57 ± 0.15*’^‡^
Cholesterol (mg/dL)	113.17 ± 10.79	178.27 ± 20.87*	281.29 ± 25.44*’^†^	216.38 ± 18.83*’^‡^	205.64 ± 19.23*’^‡^	213.11 ± 20.81*’^‡^
HDL (mg/dL)	24.22 ± 1.44	24.64 ± 1.25	24.67 ± 1.72	24.54 ± 1.73	24.37 ± 1.37	24.20 ± 1.73
LDL (mg/dL)	86.96 ± 9.34	128.98 ± 12.36*	196.67 ± 6.93*’^†^	143.27 ± 7.12*’^‡^	130.54 ± 14.72*’^‡^	148.44 ± 5.02*’^‡^
Triglyceride (mg/dL)	60.79 ± 3.41	98.75 ± 16.46*	100.82 ± 5.79*	70.40 ± 4.96^†^’^‡^	68.56 ± 7.45^†^’^‡^	97.48 ± 1.84*’^#^’^$^

Note

Values are mean ± SEM (n = 8/group).

Abbreviations: HFOA, high‐fat diet‐fed ovariectomized rats treated with atorvastatin; HFOE_2_, high‐fat diet‐fed ovariectomized rats treated with estrogen; HFOP, high‐fat diet‐fed ovariectomized rats treated with PCSK9 inhibitor; HFOV, high‐fat diet‐fed ovariectomized rats; HFS, high‐fat diet‐fed sham‐operated rats; HOMA, homeostasis model assessment; NDS, normal diet‐fed sham‐operated rats.

* *P* < 0.05 vs NDS,

^†^ *P* < 0.05 vs HFS,

^‡^ *P* < 0.05 vs HFOV,

^#^ *P* < 0.05 vs HFOA,

^$^ *P* < 0.05 vs HFOP.

### 3.2 Atorvastatin, PCSK9 inhibitor and oestrogen treatments similarly decreased PCSK9 expression, increased LDLR expression, decreased CPT1 expression, without alteration of OXPHOS expression in the liver of ovariectomized prediabetic rats

**Figure 1:**
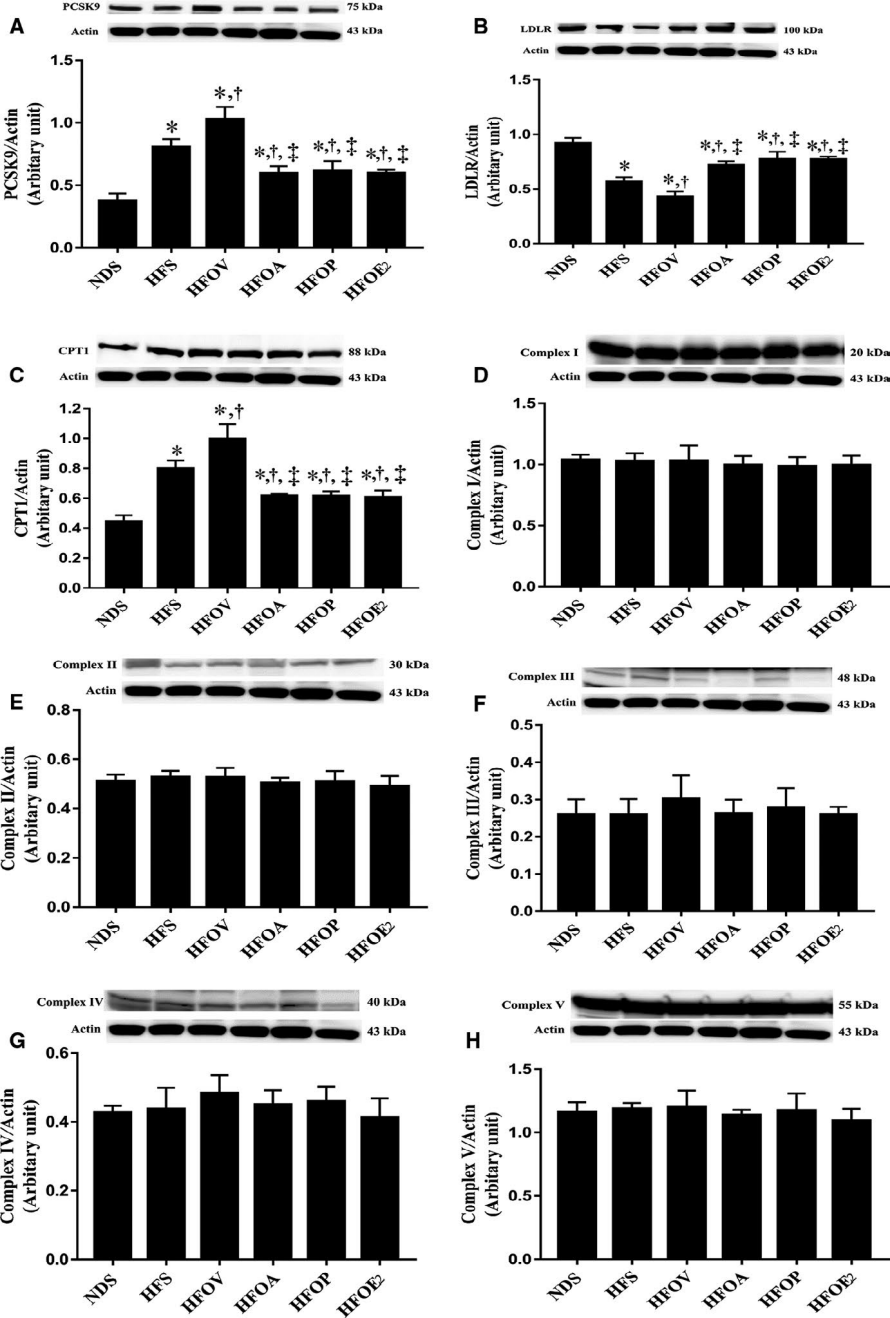
Effects of atorvastatin, PCSK9 inhibitor and oestrogen on PCSK9 expression, LDLR expression, fatty acid uptake and fatty acid oxidation in mitochondria of the liver of ovariectomized prediabetic rats. A, PCSK9 expression; B, LDLR expression; C, mitochondrial CPT1 level; D, mitochondrial complex I level. E, mitochondrial complex II level; F, mitochondrial complex III level; G, mitochondrial complex IV level; and H, mitochondrial complex V level. *P < 0.05 vs NDS, ^†^ *P* < 0.05 vs HFS, ^‡^ *P* < 0.05 vs HFOV. NDS, normal diet‐fed sham‐operated rats; HFS, high‐fat diet‐fed sham‐operated rats; HFOV, high‐fat diet‐fed ovariectomized rats; HFOA, high‐fat diet‐fed ovariectomized rats treated with atorvastatin; HFOP, high‐fat diet‐fed ovariectomized rats treated with PCSK9 inhibitor; HFOE_2_, high‐fat diet‐fed ovariectomized rats treated with oestrogen; PCSK9, proprotein convertase subtilisin/kexin type 9; LDLR, low‐density lipoprotein receptor; and CPT1, carnitine palmitoyltransferase I

The data showed that HFD‐fed rats had significantly increased PCSK9 expression and reduced LDLR expression in the liver compared to those of NDS rats (Figure 1A,B). Moreover, HFOV rats showed more aggravation in these impairments in the liver when compared with HFS rats (Figure 1A,B). HFOA, HFOP and HFOE_2_ rats exhibited a similar improvement of these impairments as indicated by markedly decreased PCSK9 expression and increased LDLR expression in the liver when compared with HFS and HFOV rats (Figure 1A,B). In addition, HFD‐fed rats had significantly increased CPT1 protein expression in the liver when compared to that of normal diet fed rats (Figure 1C). Furthermore, HFOV rats exhibited a higher level of CPT1 protein expression in the liver when compared with that of HFS rats (Figure 1C). HFOA, HFOP and HFOE_2_ rats had a similar reduction in CPT1 protein expression when compared with that of HFOV rats (Figure 1C), suggesting less fatty acid uptake into the liver. However, the expression of mitochondrial complexes I‐V proteins was not different among all six groups, even though higher fatty acid uptake was demonstrated in the HFS and HFOV groups (Figure 1D‐H). These results suggested that HFOA, HFOP and HFOE_2_ rats exhibited a higher rate of fatty acid oxidation in the liver than that of the HFS and HFOV groups. This may be a potential mechanism responsible for PCSK9 inhibitor‐induced lower level of blood triglycerides.

### 3.3 Atorvastatin, PCSK9 inhibitor and oestrogen treatments similarly decreased PCSK9 expression and increased LDLR expression in the cardiac tissue, and also similarly enhanced cardiac function in ovariectomized prediabetic rats

**Figure 2:**
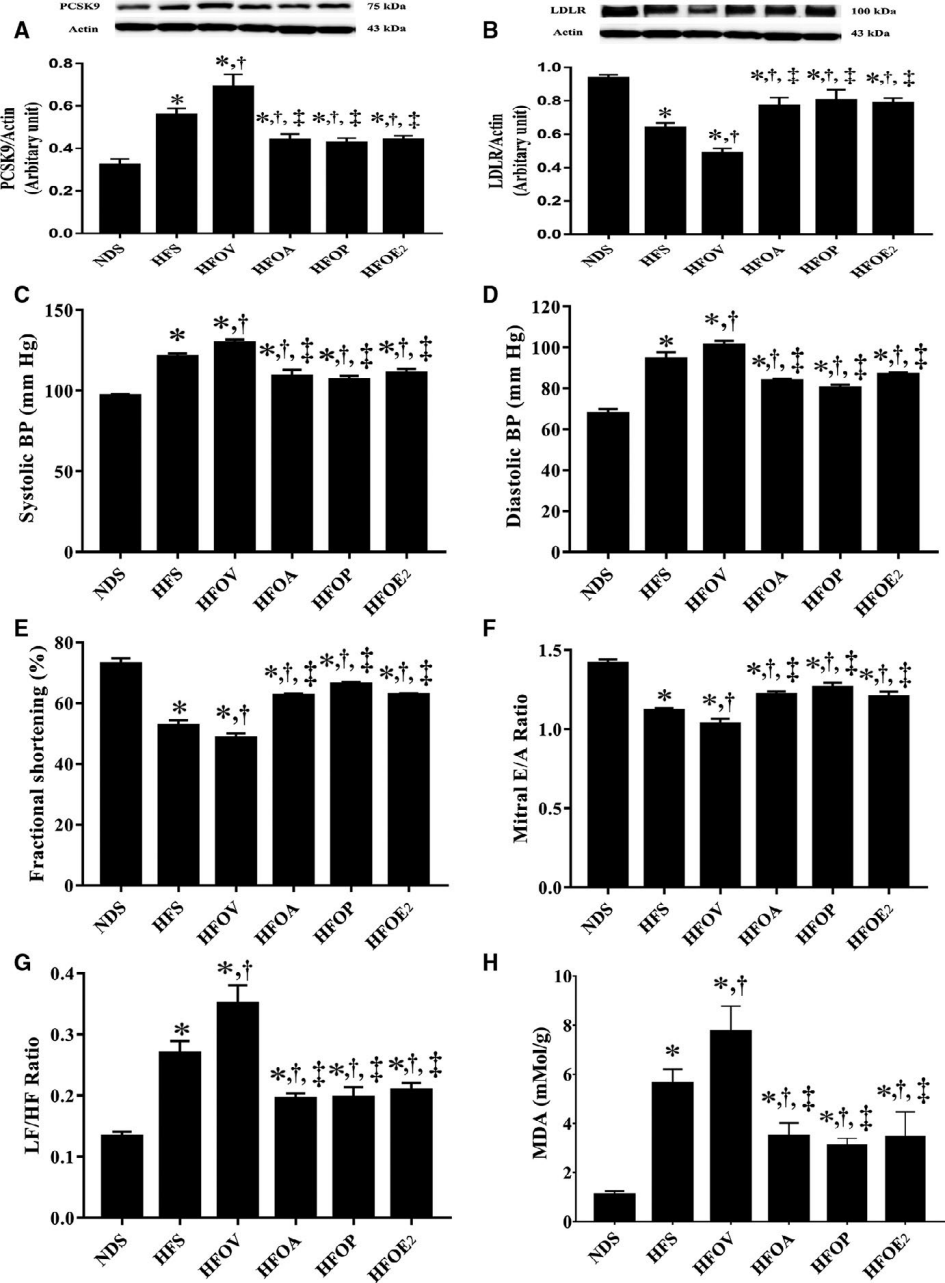
Effects of atorvastatin, PCSK9 inhibitor and oestrogen on PCSK9 expression, LDLR expression in the heart tissue and cardiac function in ovariectomized prediabetic rats. A, PCSK9 expression; B, LDLR expression; C, systolic blood pressure; D, diastolic blood pressure; E, fractional shortening (%); F, mitral E/A ratio; G, LF/HF ratio; and H, MDA. *P < 0.05 vs NDS, ^†^ *P* < 0.05 vs HFS, ^‡^ *P* < 0.05 vs HFOV. DBP, diastolic blood pressure; HFOA, high‐fat‐diet fed ovariectomized rats treated with atorvastatin; HFOE_2_, high‐fat diet‐fed ovariectomized rats treated with oestrogen; HFOP, high‐fat diet‐fed ovariectomized rats treated with PCSK9 inhibitor; HFOV, high‐fat diet‐fed ovariectomized rats; HFS, high‐fat diet‐fed sham‐operated rats; LDLR, low‐density lipoprotein receptor; LF/HF ratio, low frequency/high frequency ratio; MDA, malondialdehyde; NDS, normal‐diet fed sham‐operated rats; PCSK9, proprotein convertase subtilisin/kexin type 9; SBP, systolic blood pressure

All HFD rats had a significant increase in PCSK9 expression which was associated with the decreased LDLR expression in the heart, compared to NDS rats (Figure 2A,B). HFOV rats showed more aggravation in these impairments when compared with HFS rats (Figure 2A,B). However, HFOA, HFOP and HFOE_2_ rats had a similar reduction in these impairments as indicated by markedly decreased PCSK9 expression and increased LDLR expression when compared with HFS and HFOV rats (Figure 2A,B). In addition, SBP and DBP levels were elevated in HFD rats, when compared with NDS rats and HFOV rats had higher SBP and DBP when compared to HFS rats (Figure 2C,D). Nevertheless, HFOA, HFOP and HFOE_2_ rats had similarly improved blood pressure, in comparison with HFS and HFOV rats (Figure 2C,D). In addition, the HFD rats had reduced %FS and mitral E/A ratio when compared to NDS rats (Figure 2E,F). These impairments indicated LV dysfunction. Additionally, in HFOV rats, these impairments were aggravated when compared with HFS rats (Figure 2E,F). At the same time, atorvastatin, PCSK9 inhibitor and oestrogen treatments could similarly attenuate these impairments as indicated by increased %FS and mitral E/A ratio, when compared with HFS and HFOV rats (Figure 2E,F). The cardiac sympathovagal balance index (LF/HF ratio) was used to investigate cardiac autonomic function. HFD consumption induced cardiac sympathovagal imbalance as shown by a significant increase in LF/HF ratio when compared to NDS rats (Figure 2G). In addition, in HFOV rats these impairments were also aggravated when compared with HFS rats (Figure 2G). However, atorvastatin, PCSK9 inhibitor and oestrogen treatments attenuated these impairments to a similar extent when compared with HFS and HFOV rats (Figure 2G). Cardiac MDA level was significantly higher in HFD rats, compared to NDS rats (Figure 2H). This impairment was aggravated in HFOV rats, compared to HFS rats (Figure 2H). Notably, these impairments were similarly attenuated in HFOA, HFOP and HFOE_2_ rats (Figure 2H).

**Figure 3:**
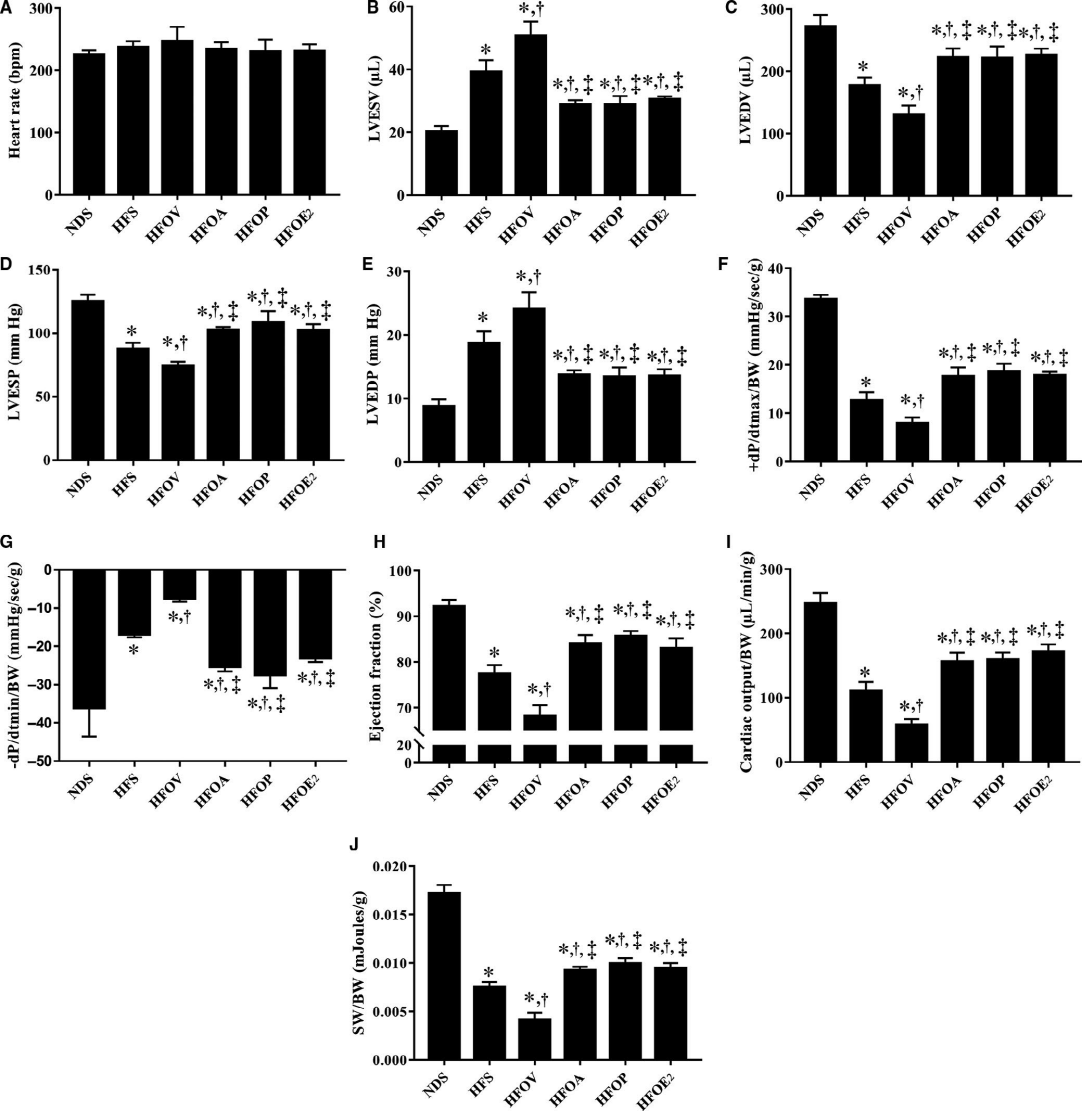
Effects of atorvastatin, PCSK9 inhibitor and oestrogen on cardiac function in ovariectomized prediabetic rats. A, Heart rate; B, LVESV; C, LVEDV; D, LVESP; E, LVEDP; F, d*P*/d*t*_max_; G, −d*P*/d*t*_min_; H, ejection fraction (%); I, cardiac output; and J, stroke work. *P < 0.05 vs NDS, ^†^ *P* < 0.05 vs HFS, ^‡^ *P* < 0.05 vs HFOV. NDS, normal diet‐fed sham‐operated rats; HFS, high‐fat diet‐fed sham‐operated rats; HFOV, high‐fat diet‐fed ovariectomized rats; HFOA, high‐fat diet‐fed ovariectomized rats treated with atorvastatin; HFOP, high‐fat diet‐fed ovariectomized rats treated with PCSK9 inhibitor; HFOE_2_, high‐fat diet‐fed ovariectomized rats treated with oestrogen; LVESV, left ventricular end‐systolic volume; LVEDV, left ventricular end‐diastolic volume; LVESP, left ventricular end‐systolic pressure; LVEDP, left ventricular end‐diastolic pressure; dP/dt_max_, maximal slope of the systolic pressure increment; dP/dt_min_, maximal slope of the diastolic pressure decrement; and CO, cardiac output

The P‐V loop was used to determine LV function. There were no differences in the heart rate values among the groups (Figure 3A). Furthermore, the data showed that HFD consumption caused a significant increase in LVESV, compared to NDS rats (Figure 3B). In addition, HFD rats had decreased LVEDV, when compared to NDS rats (Figure 3C). These impairments were also aggravated in HFOV rats, compared to HFS rats (Figure 3B‐C). Interestingly, atorvastatin, PCSK9 inhibitor and oestrogen treatments similarly attenuated these impairments when compared to HFS and HFOV rats (Figure 3B‐C). Moreover, HFD consumption caused a significant decrease in LVESP, d*P*/d*t*_max_, CO, %EF and SW, compared to NDS rats (Figure 3D‐J). In contrast, HFD rats had increased, LVEDP and d*P*/d*t*_min_, when compared to NDS rats (Figure 3E,G). These impairments, LVESV, LVEDP and d*P*/d*t*_min_, were also aggravated in HFOV rats, compared to HFS rats (Figure 3B,E,G). Interestingly, atorvastatin, PCSK9 inhibitor and oestrogen treatments similarly attenuated these impairments when compared to HFS and HFOV rats (Figure 3B‐J).

### 3.4 Atorvastatin, PCSK9 inhibitor and oestrogen treatments similarly reduced cardiac mitochondrial and dynamic dysfunction in ovariectomized prediabetic rats

**Figure 4:**
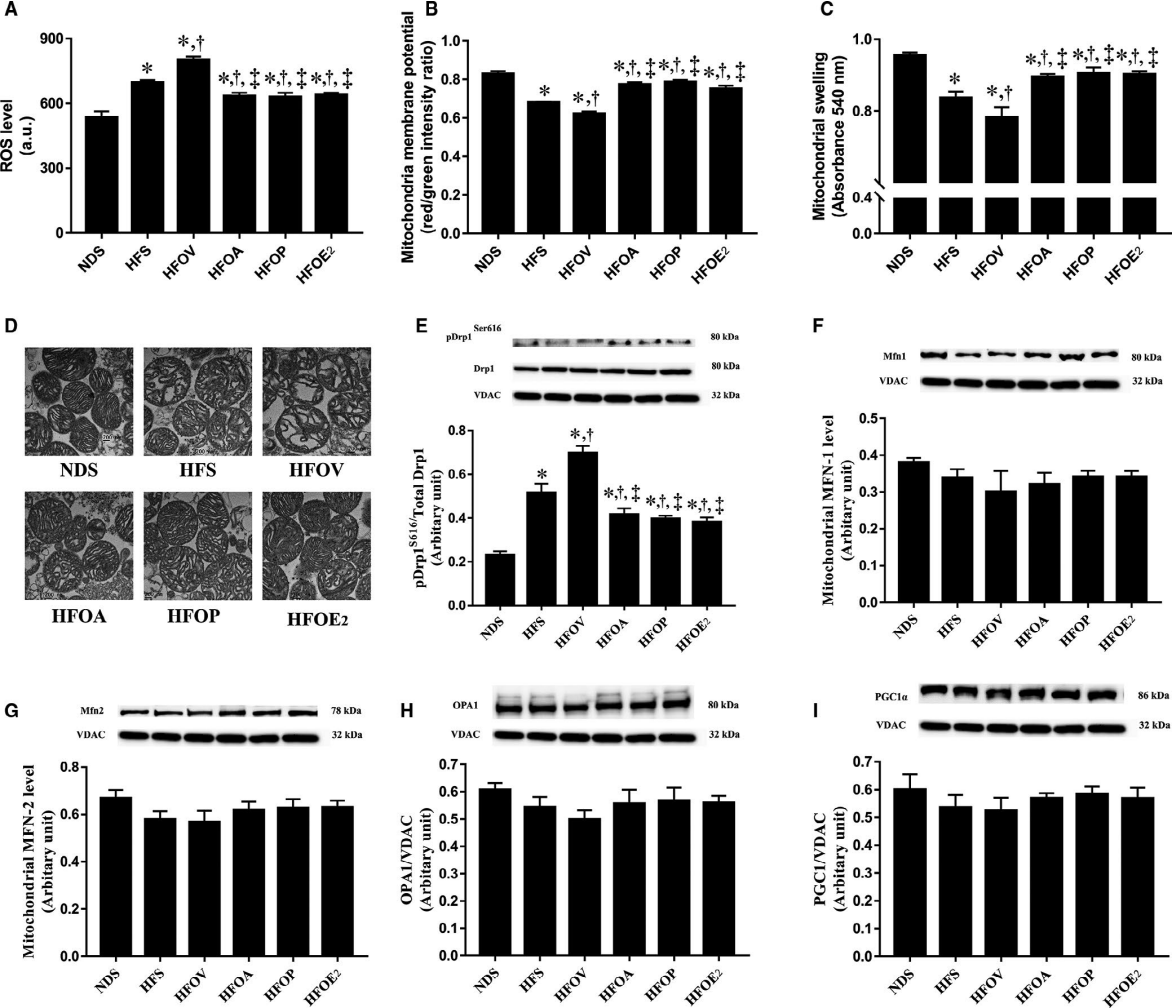
Effects of atorvastatin, PCSK9 inhibitor and oestrogen on mitochondrial function and dynamics in ovariectomized prediabetic rats. A, Cardiac mitochondrial ROS production; B, cardiac mitochondrial membrane potential; C, cardiac mitochondrial swelling; D, TEM representative images of cardiac mitochondria; E, mitochondrial Drp1 level; F, mitochondrial Mfn1 level; G, mitochondrial Mfn2 level; H, mitochondrial OPA1 level; and I, mitochondrial PGC1 level. *P < 0.05 vs NDS, ^†^ *P* < 0.05 vs HFS, ^‡^ *P* < 0.05 vs HFOV. Drp1, dynamin‐related protein 1; HFOA, high‐fat diet‐fed ovariectomized rats treated with atorvastatin; HFOE_2_, high‐fat diet‐fed ovariectomized rats treated with oestrogen; HFOP, high‐fat diet‐fed ovariectomized rats treated with PCSK9 inhibitor; HFOV, high‐fat diet‐fed ovariectomized rats; HFS, high‐fat diet‐fed sham‐operated rats; Mfn1, mitofusin 1; Mfn2, mitofusin 2; NDS, normal‐diet fed sham‐operated rats; OPA1, optic atrophy 1; PGC1, peroxisome proliferator‐activated receptor gamma coactivator 1; ROS, reactive oxygen species; TEM, transmission electron microscopy

Cardiac mitochondrial ROS production, mitochondrial membrane potential and mitochondrial swelling were measured to assess cardiac mitochondrial function. HFD rats had increased cardiac mitochondrial ROS levels, cardiac mitochondrial membrane depolarization and mitochondrial swelling in comparison with NDS rats indicating cardiac mitochondrial dysfunction (Figure 4A‐C). These impairments were aggravated in HFOV rats, when compared to HFS rats (Figure 4A‐C). Nonetheless, HFOA, HFOP and HFOE_2_ rats had similarly reduced cardiac mitochondrial ROS levels, cardiac mitochondrial membrane depolarization and mitochondrial swelling, when compared to HFS and HFOV rats (Figure 4A‐C). Mitochondria in the HFD rats showed unfolding of cristae on transmission electron micrographs when compared to NDS rats, these impairments were ameliorated to a similar extent in HFOA, HFOP and HFOE_2_ rats (Figure 4D). The level of pDrp1^Ser616^ which indicates mitochondrial fission was determined. HFD rats had significantly increased mitochondrial pDrp1^Ser616^ levels, compared to NDS rats (Figure 4E). These impairments became worse in HFOV rats, when compared to HFS rats (Figure 4E). These impairments were similarly attenuated in HFOA, HFOP and HFOE_2_ rats (Figure 4E). In contrast, there were no differences in the levels of the mitochondrial fusion proteins Mfn1, Mfn2 and OPA1, and the mitochondrial biogenesis protein PGC1 among the groups (Figure 4F‐I).

### 3.5 Atorvastatin, PCSK9 inhibitor and oestrogen treatments similarly ameliorated cardiac apoptosis in ovariectomized prediabetic rats

**Figure 5:**
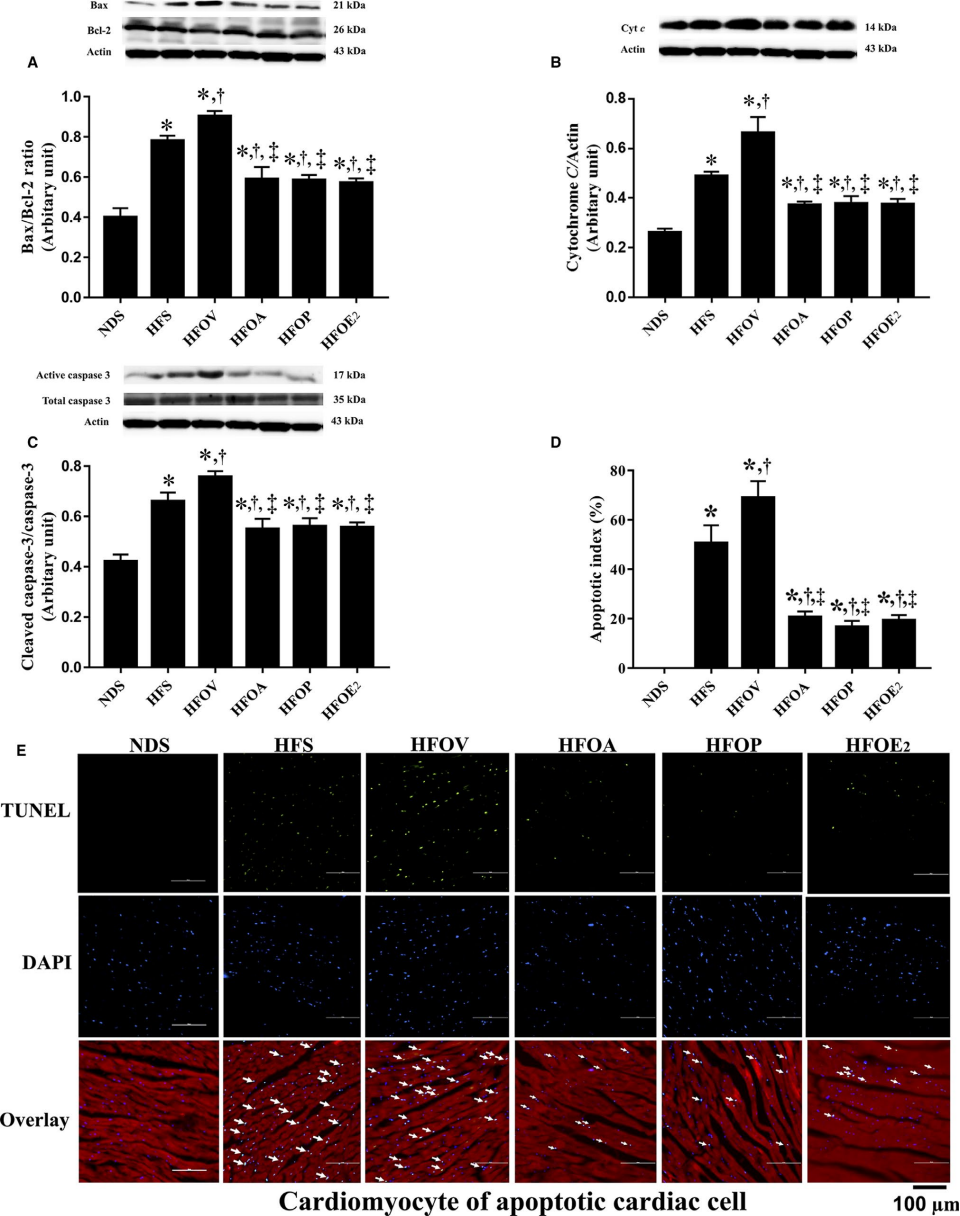
Effects of atorvastatin, PCSK9 inhibitor and oestrogen on cardiac apoptosis in ovariectomized prediabetic rats. A, Bax; B, Cytochrome *c*; C, Cleaved caspase‐3/caspase‐3; D, Apoptotic index (%); and E, Representative images of TUNEL‐positive cells. *P < 0.05 vs NDS, ^†^ *P* < 0.05 vs HFS, ^‡^ *P* < 0.05 vs HFOV. HFOA, high‐fat diet‐fed ovariectomized rats treated with atorvastatin; HFOE_2_, high‐fat diet‐fed ovariectomized rats treated with oestrogen; HFOP, high‐fat diet‐fed ovariectomized rats treated with PCSK9 inhibitor; HFOV, high‐fat diet‐fed ovariectomized rats; HFS, high‐fat diet‐fed sham‐operated rats; NDS, normal diet‐fed sham‐operated rats

Cardiac apoptosis measured using proteins Bax, Cyt *c* and Cleaved caspase‐3, and anti‐apoptotic protein Bcl‐2 was determined. HFD rats had a significant increase in the expression of Bax/Bcl‐2 ratio, Cyt *c* and Cleaved caspase‐3, compared to NDS rats (Figure 5A‐C). These impairments were also aggravated in HFOV rats, compared to HFS rats and were ameliorated to a similar extent in HFOA, HFOP and HFOE_2_ rats, compared to HFS and HFOV rats (Figure 5A‐C). HFD rats had a significant increase in the numbers of TUNEL‐positive cells when compared with NDS rats (Figure 5D‐E). However, the TUNEL‐positive cells were reduced in HFOA, HFOP and HFOE_2_ rats to a similar extent when compared to HFS and HFOV rats (Figure 5D‐E).

### 3.6 Atorvastatin, PCSK9 inhibitor and oestrogen treatments promoted intracellular calcium homeostasis to a similar extent in ovariectomized prediabetic rats

**Figure 6:**
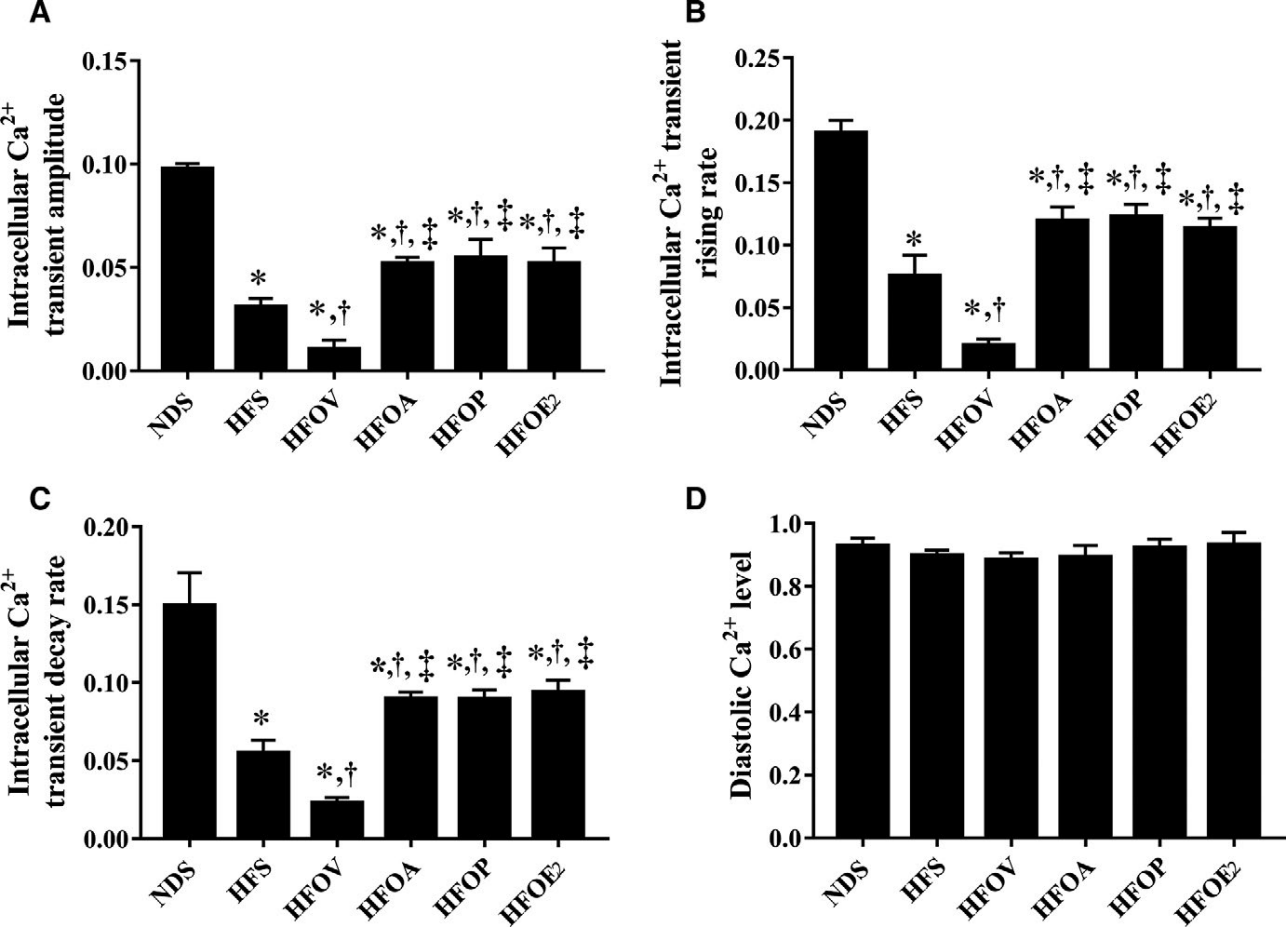
Effects of atorvastatin, PCSK9 inhibitor and oestrogen on intracellular Ca^2+^ transients in ovariectomized prediabetic rats. A, Intracellular Ca^2+^ transient amplitude; B, intracellular Ca^2+^ transient raising rate; C, intracellular Ca^2+^ transient decay rate; and D, intracellular diastolic Ca^2+^ levels. *P < 0.05 vs NDS, ^†^ *P* < 0.05 vs HFS, ^‡^ *P* < 0.05 vs HFOV. NDS, normal diet‐fed sham‐operated rats; HFS, high‐fat diet‐fed sham‐operated rats; HFOV, high‐fat diet‐fed ovariectomized rats; HFOA, high‐fat diet‐fed ovariectomized rats treated with atorvastatin; HFOP, high‐fat diet‐fed ovariectomized rats treated with PCSK9 inhibitor; HFOE_2_, high‐fat diet‐fed ovariectomized rats treated with oestrogen

Intracellular Ca^2+^ transients were used to investigate intracellular Ca^2+^ homeostasis. HFD rats showed a significant reduction in intracellular Ca^2+^ transient amplitude, intracellular Ca^2+^ transient rising rate and intracellular Ca^2+^ transient decay rate, compared to NDS rats (Figure 6A‐C). These impairments were aggravated in HFOV rats, compared to HFS rats (Figure 6A‐C). Nevertheless, these impairments were ameliorated in HFOA, HFOP and HFOE_2_ rats to similar levels, when compared with HFS and HFOV rats (Figure 6A‐C). However, there was no difference in the diastolic Ca^2+^ level among the groups (Figure 6D).

## 4 DISCUSSION

The major findings from this study clearly indicate that obese‐insulin resistance causes cardiometabolic impairment which is exemplified by metabolic disturbance, left ventricular dysfunction, cardiac mitochondrial dysfunction, cardiac apoptosis and intracellular Ca^2+^ dyshomeostasis. Furthermore, oestrogen deprivation increased these impairments in obese‐insulin resistant rats. Atorvastatin, PCSK9 inhibitor and oestrogen treatment similarly attenuated these impairments in ovariectomized prediabetic rats.

Oestrogen deprivation in models of obese‐insulin resistance or the prediabetic condition has been associated with metabolic disturbance.^36^ It is known that oestrogen can encourage energy homeostasis, increase body fat distribution, improve β‐cell function and enhance insulin sensitivity.^36^ However, because the HFS rats did not exhibit a reduction in plasma oestrogen level, it is highly suggestive that the protective effects of oestrogen are limited to the oestrogen‐deprived model. Indeed, the effects of oestrogen on non‐oestrogen‐deprived models may be harmful rather than protective.^37^, ^38^, ^39^, ^40^, ^41^ Previous studies have shown that the prevalence of metabolic syndrome has increased in post‐menopausal women.^8^, ^42^ Furthermore, our previous studies reported that obese‐insulin resistant rats with oestrogen deprivation had impaired metabolic function.^24^, ^25^ To add weight to these previous findings, the bodyweight, visceral fat, plasma glucose level, plasma insulin level, plasma cholesterol level, plasma LDL level and plasma triglyceride level in the present study were significantly higher in HFOV rats than HFS rats. Because our results demonstrated that atorvastatin and PCSK9 inhibitor could increase the rate of fatty acid oxidation in obese rats with oestrogen deprivation, this may be a potential mechanism responsible for atorvastatin‐ and PCSK9 inhibitor–induced reduction in blood triglyceride levels. Although our results also showed that oestrogen increased the rate of fatty acid oxidation in obese rats with oestrogen deprivation, blood triglyceride levels were not decreased. This could be due to the effect of oestrogen therapy on a reduction in the hepatic lipase enzyme activity and an increase in very low‐density lipoprotein (VLDL) secretion.^43^ Furthermore, it is possible that the action of PCSK9 on the levels of triglycerides is influenced by sex and sex hormone due to the different effects of sex and sex hormones on serum PCSK9 level. In fact, a previous clinical study reported that females had higher serum PCSK9 level than males.^44^ In addition, the serum testosterone level was not related to serum PCSK9 level.^44^ Moreover, testosterone replacement therapy did not affect serum PCSK9 level in males.^44^ In females, in contrast, there was a negative correlation between serum estradiol level and serum PCSK9 level, but estradiol replacement therapy did not affect PCSK9 level.^44^ These results indicated that oestrogen deprivation aggravated the metabolic impairment in obese‐insulin resistant rats.

Previous studies have reported that metabolic impairment is associated with the expression of PCSK9, which binds to LDLR and elevates its degradation, contributing to metabolic impairment.^19^, ^20^ A previous clinical study has demonstrated that post‐menopausal women with metabolic syndrome had significantly increased PCSK9 level when compared with pre‐menopausal women with metabolic syndrome.^45^ Consistent with these findings, our results showed that PCSK9 expression in the liver tissue was significantly increased in HFOV rats, when compared to HFS rats, which is associated with a reduction in LDLR expression. The results from liver tissues were in the same trend with those of cardiac tissues. Therefore, our results suggested that changes in PCSK9 and LRLR protein expression caused by high‐fat diet and oestrogen deprivation are not specific to cardiomyocyte. Our results were inconsistent with a previous study, in which they observed that ovariectomized rats had decreased expression of PCSK9 and LDLR in the liver when compared to sham‐operated rats.^46^ In that study, ovariectomized rats were fed with standard diet (12.5% energy from lipid) for 11 weeks. However, in our study, the ovariectomized rats were fed with high‐fat diet (59.28% energy from fat) for 21 weeks. Therefore, it is possible that the inconsistency between two studies is due to different dietary condition. The inhibition of PCSK9 expression could lead to increased LDLR expression and may lead to the attenuation of metabolic impairment. Atorvastatin and PCSK9 inhibitor also shared similar efficacy in attenuating the metabolic impairment in ovariectomized prediabetic rats. Atorvastatin decreased cholesterol biosynthesis via the inhibition of HMG‐CoA reductase enzyme, resulting in decreased plasma LDL level.^11^ Likewise, PCSK9 inhibitor inhibited the function of PCSK9 bound to LDLR, resulting in decreased plasma LDL levels.^19^, ^21^ The protective effects of atorvastatin and PCSK9 inhibitor are likely to be through the same mechanisms. Previous studies reported that PCSK9 inhibitor and atorvastatin could effectively reduce LDL levels in obese rats.^47^, ^48^ In addition, it has been shown that PCSK9 inhibitor effectively reduced inflammatory markers including the number of monocytes adhering and the number of T cells in the aortic root area in atherosclerotic model rats.^49^ Also, atorvastatin could reduce inflammation in the heart as indicated by increased AMPK activity and decreased NF‐κB activity in obese rats.^48^ All of these findings indicated that the protective effects of atorvastatin and PCSK9 inhibitor could be through the same mechanisms via reducing LDL levels and inflammation. Moreover, previous studies reported that PCSK9 inhibitors reduced LDL level in a dose‐dependent manner.^50^, ^51^ Therefore, it is highly possible that the effects of PCSK9 inhibitor on the heart also depend on the dose of this drug. A further study regarding the dose‐response experiment is required to support this possibility. Moreover, future studies regarding the effects of other types of statin on cardiometabolic function in ovariectomized prediabetic rats will be useful to compare the effects of various kinds of statins on cardiometabolic health in this study model. Interestingly, we found that oestrogen therapy also attenuated these metabolic impairments, the exception being triglyceride level which was significantly increased when compared to atorvastatin and PCSK9 inhibitor–treated rats. This could be due to the effect of oestrogen therapy which led to a reduction in the hepatic lipase activity and an increased synthesis of triglycerides in the liver, resulting in an increase in plasma triglyceride level.^43^, ^52^

It is known that oestrogen inhibits activity in the sympathetic nervous system, resulting in an improvement in cardiac autonomic imbalance.^53^ Oestrogen deprivation and high oxidative stress also lead to sympathetic hyperactivity and cardiac sympathovagal imbalance.^54^ This study shows that HFOV rats had increased cardiac MDA levels, indicating oxidative stress, and had an increased LF/HF ratio indicating cardiac sympathovagal imbalance. The three interventions in this study shared similarly efficacy in ameliorating the oxidative stress and cardiac autonomic imbalance. This could be due to the equal decreases in insulin resistance and oxidative stress observed in these 3 treatment groups.

Obese‐insulin resistant rats with oestrogen deprivation exhibited cardiac LV dysfunction.^55^ Our results found that atorvastatin, PCSK9 inhibitor and oestrogen similarly attenuated LV dysfunction. In addition, these three interventions also shared similar efficacy in leading to an improvement in cardiac mitochondrial function following decreased cardiac mitochondrial ROS production, mitochondrial membrane depolarization and mitochondrial swelling in ovariectomized prediabetic rats. These findings add weight to the outcome of our previous study which also found an imbalance in mitochondrial dynamics including increased mitochondrial fission in obese‐insulin resistant rats with oestrogen deprivation.^55^ Consistent with these findings, we found that HFOV rats had a significantly increase in the pDrp1 at serine 616 which indicates mitochondrial fission. Atorvastatin, PCSK9 inhibitor and oestrogen equally attenuated mitochondrial dynamic imbalance. In contrast, cardiac mitochondrial fusion was not altered in these rats. This is consistent with our previous study which showed that 13 weeks of HFD consumption with oestrogen deprivation increased the level of mitochondrial fission proteins without any change in the level of mitochondrial fusion proteins.^55^ However, the reduction in mitochondrial fusion proteins did occur in rats on a HFD for 40 weeks; therefore, it can be postulated that mitochondrial fusion protein alteration occurs after longer term HFD consumption.^44^ In addition, mitochondria undergoing the fission process, concurrently with the activation of Bax, results in increased Bax activated mitochondrial release of Cyt *c*, which leads to caspase 3 activation and cardiomyocyte apoptosis.^56^, ^57^ However, atorvastatin, PCSK9 inhibitor and oestrogen effectively decreased Bax, Cyt *c*, and Cleaved caspase‐3 levels, and cardiac cell apoptosis.

**Figure 7:**
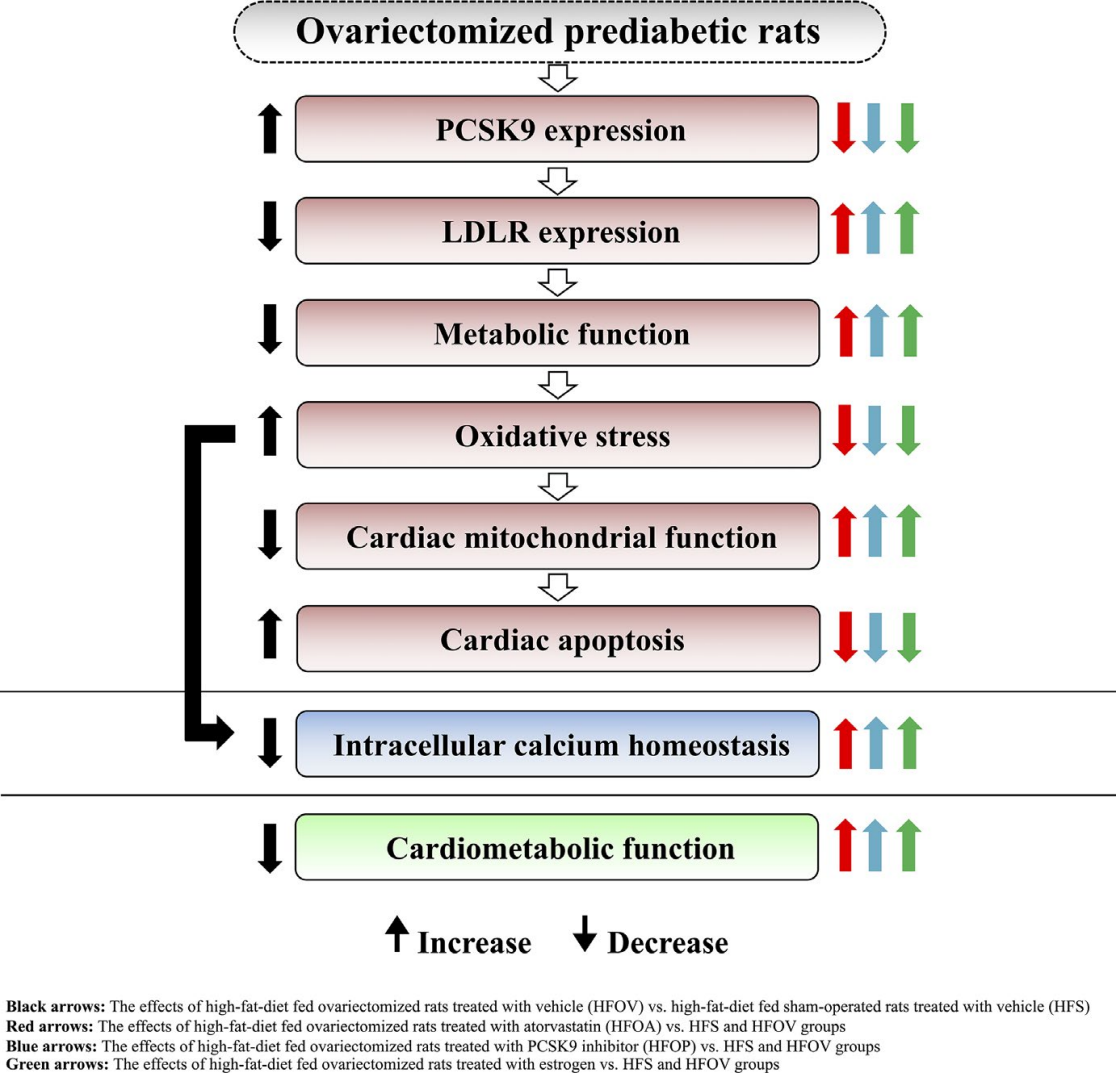
Summary of atorvastatin, PCSK9 inhibitor and oestrogen treatment on cardiometabolic function in ovariectomized prediabetic rat

Our previous study reported that obese‐insulin resistant rats with oestrogen deprivation have intracellular Ca^2+^ transient dyshomeostasis following a reduction in intracellular Ca^2+^ transient amplitude, intracellular Ca^2+^ transient rising rate and intracellular Ca^2+^ transient decay rate, which lead to cardiac contractile dysfunction.^58^ Our results showed that atorvastatin, PCSK9 inhibitor and oestrogen effectively decreased intracellular Ca^2+^ transient dyshomeostasis, as shown by similar increases in intracellular Ca^2+^ transient amplitude, intracellular Ca^2+^ transient rising rate and intracellular Ca^2+^ transient decay rate. All of these benefits could lead to the attenuation of cardiac dysfunction. All of the findings resulting in the improvement of cardiometabolic function are summarized in Figure 7.

## 5 CONCLUSION

Our study is the first to describe the comparative efficacy of atorvastatin, PCSK9 inhibitor and oestrogen on cardiometabolic function in ovariectomized prediabetic rats. Our findings indicate that these three interventions shared similar efficacy in improving cardiometabolic function in these models.

## CONFLICT OF INTEREST

The authors declare that they have no competing interests.

## AUTHOR CONTRIBUTION

Patchareeya Amput: Data curation (lead); Formal analysis (lead); Methodology (lead); Writing‐original draft (lead). Siripong Palee: Data curation (supporting); Formal analysis (supporting); Methodology (supporting); Validation (supporting). Busarin Arunsak: Formal analysis (supporting); Investigation (supporting); Methodology (supporting); Validation (supporting). Wasana Pratchayasakul: Data curation (supporting); Formal analysis (supporting); Investigation (supporting); Methodology (supporting); Validation (supporting). Chanisa Thonusin: Formal analysis (supporting); Methodology (supporting); Validation (supporting); Visualization (supporting); Writing‐review & editing (supporting). Sasiwan Kerdphoo: Formal analysis (supporting); Investigation (supporting); Methodology (supporting); Validation (supporting). Thidarat Jaiwongkam: Formal analysis (supporting); Investigation (supporting); Methodology (supporting); Validation (supporting); Visualization (supporting). Siriporn Chattipakorn: Conceptualization (supporting); Data curation (supporting); Formal analysis (supporting); Funding acquisition (equal); Investigation (supporting); Methodology (supporting); Supervision (supporting); Validation (supporting); Visualization (supporting); Writing‐review & editing (supporting). Nipon Chattipakorn: Conceptualization (lead); Data curation (supporting); Formal analysis (supporting); Funding acquisition (lead); Investigation (supporting); Methodology (supporting); Project administration (lead); Resources (lead); Supervision (lead); Validation (supporting); Visualization (supporting); Writing‐review & editing (lead).

## Data Availability

The data that support the findings of this study are available from the corresponding author upon reasonable request.
